# Variation in Communication and Family Visiting Policies in Italian Intensive Care Units during the COVID-19 Pandemic: A Secondary Analysis of the COVISIT International Survey

**DOI:** 10.3390/healthcare11050669

**Published:** 2023-02-24

**Authors:** Barbara Simone, Mariachiara Ippolito, Pasquale Iozzo, Francesco Zuccaro, Antonino Giarratano, Maurizio Cecconi, Alexis Tabah, Andrea Cortegiani

**Affiliations:** 1 Department of Surgical, Oncological and Oral Science, University of Palermo, Via Liborio Giuffrè 5, 90127 Palermo, Italy; 2 Department of Anaesthesia, Intensive Care and Emergency, University Hospital Policlinico Paolo Giaccone, 90127 Palermo, Italy; 3 Department of Anesthesia and Intensive Care, Ospedale Madonna delle Grazie, Azienda Sanitaria Matera, 75100 Matera, Italy; 4 Department of Biomedical Sciences, Humanitas University, Via Rita Levi Montalcini 4, Pieve Emanuele, 20072 Milan, Italy; 5 Department of Anesthesia and Intensive Care, IRCCS Humanitas Research Hospital, Via Manzoni 56, Rozzano, 20089 Milan, Italy; 6 Intensive Care Unit, Redcliffe Hospital, Metro North Hospital and Health Services, Redcliffe, QLD 4020, Australia; 7 School of Clinical Sciences, Queensland University of Technology, Brisbane, QLD 4000, Australia; 8 Antimicrobial Optimisation Group, UQ Centre for Clinical Research, The University of Queensland, Brisbane, QLD 4029, Australia

**Keywords:** communication, visiting policies, Italy, ICU, COVID-19

## Abstract

Background: During COVID-19 pandemic, restrictions to in-person visiting of caregivers to patients admitted to intensive care units (ICU) were applied in many countries. Our aim was to describe the variations in communication and family visiting policies in Italian ICUs during the pandemic. Methods: A secondary analysis from the COVISIT international survey was conducted, focusing on data from Italy. Results: Italian ICUs provided 118 (18%) responses out of 667 responses collected worldwide. A total of 12 Italian ICUs were at the peak of COVID-19 admissions at the time of the survey and 42/118 had 90% or more of patients admitted to ICU affected by COVID-19. During the COVID-19 peak, 74% of Italian ICUs adopted a no-in-person-visiting policy. This remained the most common strategy (67%) at the time of the survey. Information to families was provided by regular phone calls (81% in Italy versus 47% for the rest of the world). Virtual visiting was available for 69% and most commonly performed using devices provided by the ICU (71% in Italy versus 36% outside Italy). Conclusion: Our study showed that restrictions to the ICU applied during the COVID-19 pandemic were still in use at the time of the survey. The main means of communication with caregivers were telephone and virtual meetings.

## 1. Introduction

In Italy, after the first two COVID-19 cases were confirmed and a state of emergency declared, several measures were taken in order to limit the spread of contagions across the national territory: the suspension of educational activities in all schools of each degree and university, the establishment of remote working when applicable, and the prohibition of access by relatives and visitors to health facilities [1]. Since then, to deal with the emergency, extraordinary personnel recruitment procedures have been used with the possibility of hiring newly graduated doctors, retired personnel, and senior resident doctors, and to allocate physicians in wards outside their main specialties. Funds have therefore been allocated for the construction of new infrastructures and to increase the number of hospital and intensive care unit (ICU) beds. Moreover, elective surgeries, admission to ordinary wards, and outpatient visits were halted, and several ordinary wards and ICUs were repurposed for COVID-19 patients. In some cases, even operating rooms were set up as ICUs to increase bed availability. Although there were no national formal criteria for admission of patients in COVID-19 ICUs, a multidisciplinary document issued by relevant national societies (SIAARTI-SIMLA) on decisions in case of discrepancies between care needs and resources during the pandemic stated that the triage to ICU admission should be oriented to ensure organ support treatments to as many patients as possible who may benefit from them. Indeed, “the decision should follow the full assessment of each patient, taking into account comorbidities, previous functional status and frailty, current clinical condition, likely impact of intensive treatment, and the patient’s wishes. Age should be considered as part of the global assessment of the patient” [2]. During the COVID-19 pandemic, mandatory restrictions to in-person visiting of caregivers and family to patients admitted to ICU were also applied, to limit in-hospital contagions among patients, relatives, and healthcare providers, to avoid shortages of personal protective equipment supplies, to improve patient safety [3,4], and to manage the huge workload above usual running capacity [5]. The emergency caused such decisions to be applied despite the positive effects of relatives visiting being well-known, not just for patients in terms of clinical conditions (e.g., possible reduction in the incidence of delirium and post-traumatic stress disorder) [6,7,8,9], but also for relatives (e.g., reducing burden and anxiety) [8,10]. Indeed, poor communication with ICU staff, visiting restrictions, and lack of information regarding ICU care of relatives were identified as risk factors of Post-Intensive Care Syndrome family (PICS-F) [11]. Moreover, a high prevalence of PTSD symptoms has been specifically observed in persons having a family member with COVID-19 and admitted to an ICU, and symptoms have been associated with different demographic characteristics and comorbidities (e.g., ethnicity, female gender), with distrust of practitioners described as highly prevalent among those with higher PTSD scores [12]. Thus, the extent of the burden of restrictions to in-person visiting may have been variable in different settings and populations of caregivers and influenced by many factors.

COVISIT was an international survey conducted in 2021, aiming to describe policies and methods of communication with ICU patients’ relatives prior to and during the COVID-19 pandemic [13]. Although Italy was the first European country to be hit by the COVID-19 pandemic, little is known about the visiting and communication policies in Italian ICUs during this period [14].

Our aim was to describe visiting policies and methods of communication used in Italy with relatives of ICU patients prior to and during the COVID-19 pandemic.

## 2. Materials and Methods

For the purpose of this descriptive cross-sectional study, we used data from the COVISIT [13] database and performed a secondary analysis focusing on data collected from respondents based in Italy. COVISIT was an international survey conducted between March and July 2021, investigating ICU visiting policies and communication strategies at three timepoints: before COVID-19, at peak ICU admissions (highest number of COVID-19 patients in the ICU prior to survey completion), and at the time of survey completion (also referred to as “post-pandemic peak” through the text). For ICUs that were at the peak at the time of survey response, peak data were equal to the time of the survey. The survey was conducted in 667 ICUs and was promoted by the European Society of Intensive Care Medicine (ESICM), and endorsed by several other societies worldwide, including SIAARTI (Società Italiana di Anestesia Analgesia Rianimazione e Terapia Intensiva) for Italy. The full methods and report of the original study are publicly available in the published article [13].

In brief, a questionnaire was administered through a web-based platform to healthcare professionals working in ICUs worldwide, following the plan to analyze one response per ICU: duplicate responses were identified manually and excluded following a stipulated hierarchy (medical director, nurse unit manager or nursing director, senior medical role, senior nursing role, medical other, nursing other, administrative role, other). The survey contained introductory questions to identify the ICU (e.g., answers about country, town, name of the hospital, and name of the ICU), aiming at verifying the answers validity and allowing for duplicate cleaning, but not further analyzed to ensure anonymity. The questionnaire investigated 32 items, with questions regarding three domains: (1) staffing ratios and visiting hours; (2) how visiting and family communication policies were developed and modified over the pandemic; (3) communication strategies and use of virtual visiting. These were examined focusing on the time of the survey, while domain (1) was investigated over three timepoints—before COVID-19, at peak ICU admissions, and at the time of survey completion—to provide baseline data. The survey also collected data on the characteristics of the ICUs where respondents worked.

The questionnaire was available online and distributed as a link, sent to eligible participants through mailing lists and advertised through social media by the endorsing societies (see Acknowledgment section for the full list), including SIAARTI for Italy. The survey was available in four different languages, including Italian.

### Statistical Analysis

Data cleaning was already performed for the original study, and, for the purpose of this secondary analysis, the cleaned version of the original database was used. Full description of data cleaning and categorizations is provided in the original study [13]. Values were reported for available responses for each variable at the relevant timepoint. The number of missing data are shown in the tables. We did not perform any imputation for missing data. At the original study analyses stage, duplicates had already been excluded following a pre-specified order according to the respondent role, i.e., using the following hierarchy: medical director, nurse unit manager or nursing director, senior medical role, senior nursing role, medical other, nursing other, administrative role, other.

Data were then analyzed for the purpose of our secondary analysis using descriptive statistics and presented as median and interquartile range or percentages and fractions, as appropriate. Data were then graphically presented using tables and graphs. We performed the chi-square test to detect differences in the investigated ICUs’ characteristics between the two groups (e.g., Italy vs. outside of Italy). Statistical significance was defined as *p* < 0.05. Data analysis was performed by BS with input from MI and AC, using Microsoft Excel (version 16.66.1; Microsoft Corporation, Redmond, CA, USA).

## 3. Results

A total of 667 valid responses were collected worldwide, most (52%) from Europe and Central Asia. In Italy, a total of 118 (18%) ICUs were surveyed.

At the time of the survey, 12% of Italian ICUs were at peak COVID-19 admissions. For the 88% of other respondents, the time from the peak of COVID-19 admissions to the time of the survey was a mean of 8 months (range: 1 to 14 months). In Italy, 97 out of the 118 (82%) surveyed ICUs already existed before COVID-19, while 21 (18%) were built specifically for the COVID-19 pandemic. Most of the responses (92%, 108/118) were from ICUs in public hospitals, with 3% (3/118), 5% (6/118), and 1% (1/118) from private for-profit, private not-for-profit, and mixed funding hospitals, respectively. Hospital size ranged from fewer than 250 beds (32%, 30/95) to more than 1000 beds (20%, 19/95), with 25% (24/95) having 250 to 499 beds and 23% (22/95) having 500 to 999 beds.

### 3.1. Staffing and Logistic Characteristics

Staffing and organizational characteristics of the Italian ICUs at the time of the survey are shown in Table 1, along with those from other countries.

At the time of the survey, the 43% of respondents in Italy, and 60% outside, declared to have had fewer than 25 of patients affected by COVID-19 among those admitted to their ICUs. During the COVID-19 peak, 53% of the surveyed Italian ICUs reported that 90% or more of the admitted patients were affected by COVID-19 during the peak (Table S1).

At the time of the survey, 40% of the surveyed Italian ICUs had 9 to 16 beds. Approximately half of the respondent ICUs had a distribution of senior doctor-to-patient ratio between 1:6 and 1:10. Thirty-four (45%) had a junior doctor-to-patient ratio of 1:6 to 1:10. Most (74/108, 69%) had one nurse every two patients.

Statistically significant differences were found in the distributions of answers assessing the number of patients with COVID-19 admitted to ICU, junior doctor-to-patient ratio, and nurse-to-patient ratio when comparing Italian answers to those from other countries.

### 3.2. Visiting Policies

**Figure 1:**
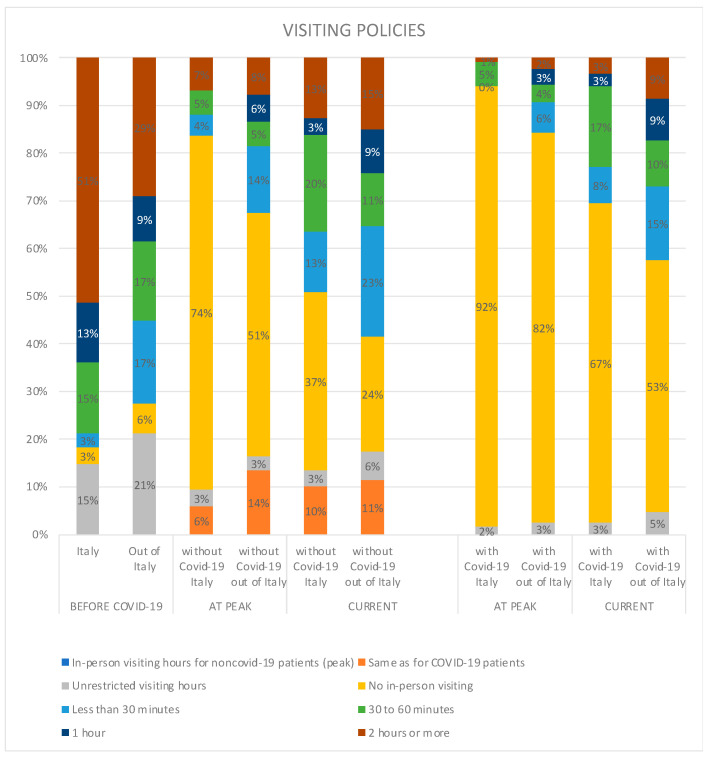
Comparison of visiting policies in Italy and outside Italy over three timepoints (before COVID-19, at peak, and at the time of the survey) according to COVID-19 status of patients.

Before the COVID-19 pandemic, most ICUs allowed 2 h or more of visiting in Italy (51%), and 29% elsewhere (Figure 1). Unrestricted visiting hours were offered by 15% in Italy and 21% outside of Italy. On the other hand, 3% of the surveyed ICUs in Italy and 6% outside Italy did not allow in-person visiting at all. At the peak of COVID-19, a no-in-person-visiting policy had been implemented by 92% for patients with COVID-19, and 74% for patients without COVID-19. Similar policies were reported by ICUs around the world, with, respectively, 82% and 51% having no-in-person-visiting policies. Only 6% of surveyed ICUs in Italy and 14% outside Italy applied the same visiting policy, irrespective of COVID-19 diagnosis. At the time of the survey, the most common strategy remained no in-person visiting, both in Italy and outside Italy, for COVID-19 (67%, 79/118 in Italy; 53%, 290/549 outside Italy) and non-COVID-19 (37%, 44/118 in Italy; 24% 132/549 outside Italy) patients.

As shown in Table 2, at the time of the survey, in Italy and worldwide, most ICUs (64% in Italy and 68% outside of Italy) had written visiting policies designed or revised for the COVID-19 pandemic, despite the government not directly mandating any restriction policy in 43% of the cases in Italy and 48% outside Italy.

**Figure 2:**
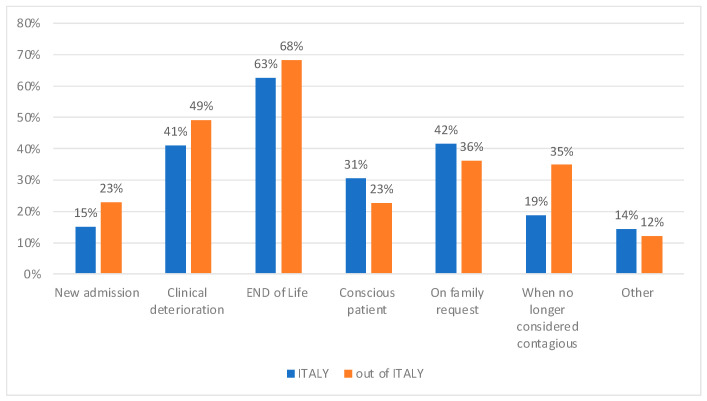
Reasons for allowing visiting when it would have been restricted by the set policies.

Visiting policies applied the same rules for all the hospital wards in most of the cases (52%) in Italy, and 42% outside of Italy. Despite that, ICU visiting policies could be, even temporarily, changed for specific patients or situations, on the basis of decision by the ICU medical director (63% in Italy; 40% outside of Italy), doctors (33% in Italy; 48% outside of Italy), or the ICU nursing director (17% in Italy; 17% outside Italy). Indeed, most of the respondents (87% in Italy and 82% outside of Italy) declared that a more liberal policy was offered to relatives of ICU patients, allowing them to stay at the bedside more often or for longer periods, and that this occurred in cases of clinical deterioration and end of life, followed by family request (Figure 2).

### 3.3. Communication Strategies

Seven questions investigated the use of communication methods with relatives during ICU stay at the time of the survey (Table 3). The 69% (82/118) of Italian ICUs did not use any booklet or webpage with information about COVID-19. General or daily updates were given outside the clinical area of ICUs (19%, 23/118) in Italy versus 38% (207/549) outside of Italy, when in person. Otherwise, information was provided by phone with regular calls to family (81%, 96/118 in Italy versus 47%, 257/549 outside of Italy), also including discussions regarding prognosis, treatment plans, or end-of-life care (70%, 83/118 in Italy versus 41%, 223/549 outside of Italy). Virtual visiting was used but not protocolized in 69% of the ICUs in Italy and 44% outside Italy, or performed daily for most patients (33%, 33/118 in Italy versus 24%, 78/549 outside of Italy), or performed several times per week (30%, 30/118 in Italy versus 30%, 96/549 outside of Italy).

When virtual visiting was organized, it was most frequently upon relatives’ request (50%, 59/118 in Italy versus 30%, 164/549 outside Italy), or without an appointment (36%, 43/118 in Italy versus 24%, 133/549 outside Italy). Virtual visiting was most commonly performed using dedicated devices made available by the ICU (71%, 84/118 in Italy versus 36%, 195/549 outside Italy), or with personal devices provided by patients or their relatives (25%, 30/118 in Italy versus 27%, 150/549 outside Italy).

## 4. Discussion

Our study described policies and methods of communication with relatives of patients admitted to ICU in Italy in the periods prior to, during, and after the COVID-19 pandemic. At the time of the survey, the most common strategy was no in-person visiting, even after the start of the vaccination campaign [15]. This was the most important finding of the study, reflecting that a full restoration of previously adopted visiting policies did not occur at the time of the survey, despite the peak of COVID-19 having passed for 88% of the responding ICUs.

Nevertheless, many ICUs reported more liberal policies for particular cases, such as end-of-life and clinical deterioration, showing that the emotion and value of staying at the bedside of a sick relative, communication with relatives, and setting goals of care ensuring dignity in death and decision-making power [16] were recognized by healthcare providers as a valid trigger for modification of policy, even in the context of the pandemic. Another striking finding was that the need for a drastic and rapid reorganization prevented the structured and fast implementation of communication methods. Most Italian respondents declared not having any dedicated COVID-19 information booklet or webpage for visitors of ICU patients, and that the telephone remained often the main and only means of communication, with virtual visiting most often conducted upon request from the relatives. Concordantly, data from Italy and other countries showed that videocalls and virtual visiting became a usual means of communication during pandemic restrictions [13,17,18]. Our data confirm that the COVID-19 pandemic has been a challenge for modern healthcare systems, not only because of the need for advanced life-sustaining treatments in intensive care units, often with an imbalance between resources and needs, but also due to the huge impact on communication and visiting policies and to the potentially distressing impact these issues have had on many people. It is indeed known that not saying goodbye to a dying relative may be associated with increased psychological burden [19] and symptoms of PTSD in family members of ICU patients with COVID-19 [12], and growing evidence has shown associations between visit restrictions and detrimental effect on patients’ health, the health and wellbeing of family members, and the provision of care [20] in most settings of care, including the ICU [21].

Furthermore, it has been widely discussed that an ‘‘open’’ visiting policy may contribute to reducing stress and anxiety for relatives of ICU patients [22,23], and in 2013 [24] the Italian National Committee for Bioethics (INCB) had already highlighted the importance for ICUs to be organized towards promoting the right of patients admitted to the ICU to stay in the presence of family members or a dear person. The pandemic challenged these opportunities and created an unprecedented situation, forcing institutions and governments to provide regulations for visit restrictions on the basis of weak scientific evidence [20,21].

The dimension of the problem and its perceived impact in Italy was also witnessed by the increasing interest across the scientific community. Indeed, a national, multicenter survey was also conducted [14] between 24 February and 31 May 2020, describing the changes in hospital/ICU organization and ICU visiting/communication habits during the first COVID-19 pandemic wave in Italy, and comparing them with pre-pandemic data. Despite similar results, the study differs from our analysis as it focused on the first pandemic wave and did not provide data on subsequent periods. Overall, the literature seems concordant in describing that relatives of ICU patients had no access to the ICU during the first COVID-19 pandemic wave, and daily remote communications served as a surrogate for family meetings. Questions remain on the appropriateness of video and virtual visits to show patients’ clinical pictures, especially those in critical conditions or perimortem. The potential detrimental phycological consequences of seeing loved ones in different shape or with different facies than in previous memories (e.g., face and body alterations due to consequences of the disease and care) must be balanced with the opportunity for relatives to “stay in touch” and say goodbye, with important consequences for grieving.

The importance of family visiting is well-known, and several studies have debated the benefits of opening the ICU to relatives in terms of physiological benefits for both patients and families, such as reduced blood pressure and heart rate [6], reduced occurrence and length of delirium and ICU stay [25], and also, for family, reducing burden and anxiety [10], and improving satisfaction and emotional well-being [26].

Rose L. et al. described the potential benefits of providing virtual visiting instead of no visiting through their cross-sectional study led in UK hospitals with at least one ICU [18]. In line with our study, they report that all (100%) of the interviewed hospitals changed their visiting policy and that, when in-person family visiting was allowed in particular circumstances, the most common reason was “End of Life”. Interestingly, 34% of surveyed hospitals implemented a dedicated ICU communication/family liaison team and virtual visiting was found to reduce patient psychological distress (78%) and increase staff morale (68%).

The impact of different communication methods and visiting policies has been variously described in the literature. In a cross-sectional survey conducted by Chanchalani G. et al., 92% of ICUs in South Asia and the Middle East had significant changes in the daily visiting duration in COVID ICUs, limiting visiting hours, but most ICUs (65.3%) used fixed visiting hours, while only 32.3% of ICUs reported a “no-visitor” policy [27]. These data are very interesting if compared to the Italian data reported in our survey, where most ICUs (92%) reported a “no-visitor” policy for COVID-19 patients during the pandemic peak, and also for non-COVID-19 patients (74%), showing more liberal policies regarding patient visits were adopted elsewhere.

A cross-sectional study conducted by Rodriguez-Ruiz E. et al. on 270 family members of patients in two Spanish ICUs studied the impact of different visiting policies on family satisfaction before and during COVID-19: before COVID-19, one had restricted visiting of 1 h twice a day at mealtimes, while the other had an open visiting policy; after COVID-19 restriction, both had 1 h a day for only one relative. They demonstrated that restriction impacted family satisfaction, in particular in the ICU with previous open visiting policies [28]. A qualitative study conducted in France by Kentish-Barnes N. et al. demonstrated, through an interview with the families of patients who died of severe COVID-19 in ICU, the dramatic impact on the experiences because of the interruptions in the relationship with their loved ones and disruptions in end-of-life rituals [29]. These studies confirm that other European countries have applied restrictions and that these have aroused scientific interest, probably based on a large emotional impact and both studies agree on the need for a reopening towards the presence of family members.

Interesting speculations are also possible on the Italian data and the original international COVISIT study. First of all, the distribution of resources may have been different: for example, the nurse-to-patient 1:1 ratio has been reported as decreased from 17% in pre-pandemic times to 15% at peak and at the time of the survey in the international original study. In Italy, this ratio was present in 1% of the ICUs at the time of the survey and never even found in the pre-pandemic period or at its peak (see File S1 at Supplementary Material). This was also confirmed and found significant by our quantitative analysis (Italy vs. outside of Italy, see Table 1). Moreover, no restricting visiting policy in wards was reported in 11% of cases in the COVISIT survey at the time of the survey, while in our data just 3% of the ICUs did not adopt restrictions. Similar attitudes were registered regarding methods of communication to family, as the main support was the phone and family were called at regular intervals by ICU staff both in the original study (55%) and in our findings (81%).

Despite our study not being aimed at analyzing the differences among practice at a national level, the interpretation of our data, once put in context with data from other countries, may offer insights on potential reasons for reasonable geographical differences in restrictions and communication policies, e.g., the different surge of the pandemic on healthcare systems in countries, different national guidelines, regulations or documents issued by national societies that may have influenced the practice locally, and different decisions, management, and communication to patients and relatives about end-of-life and life-sustaining therapies worldwide, potentially due to ethical, cultural, religious, and logistic factors [30]. We can only speculate on such potential causes since the design of the main analysis and this secondary analysis of the dataset from the COVISIT survey did not permit a precise evaluation of causalities and associations.

However, we believe that our data and the overall results from the COVISIT dataset may help understanding of local applications of visit restrictions, exceptions, and communication strategies held and how they had been modified during the course of the pandemic. In the case of further presumed need for restricting visits in the ICU, these data may help as background to explore other levels of regulated restrictions (e.g., no restriction at all, single visits during the ICU stay, or just in case) and make comparisons and eventual associations with patients’ and families’ outcomes.

### Limitations

In addition to the limits of the original study [13], we acknowledge that the survey was not aimed to reach homogeneous geographic diffusion within countries, including Italy. Indeed, the participation of ICUs was on a voluntary basis. Thus, the inclusion was not systematic. For these reasons, regional differences in data provided by Italian ICUs cannot be explored. Furthermore, at the protocol stage, we did not plan quantitative analyses to compare the restrictions and communication methods adopted by the different countries, and therefore the study was descriptive in nature; thus, we were not able to investigate associations and cause/effect relationships or test associations between characteristics of ICUs and restrictions or effects.

## 5. Conclusions

Mandatory restrictions to ICU visiting policies were applied during the COVID-19 pandemic in Italy, and pre-pandemic policies were not restored from the peak of COVID-19 to the time of the survey. The main means of communication adopted during the pandemic were telephone and virtual meetings. Further studies may aim at investigating measures to optimize communication strategies with family members and their involvement to reduce the risk of feelings of separation and complicated grief in case of a need for visiting restrictions.

## Figures and Tables

**Table 1:** Characteristics of ICUs of respondents in Italy and outside of Italy at the time of the survey.

Characteristics	Italy *n* = 117	Outside of Italy *n* = 547	
ICU patients with COVID-19 (%)			*p* = <0.001
Fewer than 25	50 (43%)	329 (60%)	
25 to 49	4 (3%)	68 (12%)	
50 to 89	21 (18%)	87 (16%)	
90 or more	42 (36%)	63 (12%)	
Total number of ICU beds *			*p* = 0.080
1 to 8	29 (25%)	123 (23%)	
9 to 16	47 (40%)	174 (32%)	
17 to 24	22 (19%)	90 (17%)	
25 to 40	12 (10%)	80 (15%)	
More than 40	7 (6%)	74 (14%)	
Senior doctor-to-patient ratio **			*p* = 0.062
Less than 1:6	41 (39%)	184 (43%)	
1:6 to 1:10	54 (51%)	171 (40%)	
More than 1:10	10 (10%)	70 (16%)	
Junior doctor-to-patient ratio ***			*p* = 0.014
Less than 1:6	33 (43%)	236 (62%)	
1:6 to 1:10	34 (45%)	110 (29%)	
More than 1:10	9 (12%)	32 (8%)	
Nurse-to-patient ratio ****			*p* = <0.001
1:1	1 (1%)	85 (19%)	
1:2	74 (69%)	182 (41%)	
1:3	27 (25%)	119 (27%)	
More than 1:3	6 (6%)	61 (14%)	

* Answers to this question were provided by *n* = 541 respondents outside of Italy. ** Answers to this question were provided by *n* = 105 respondents from Italy and *n* = 425 outside of Italy. *** Answers to this question were provided by *n* = 76 respondents from Italy and *n* = 378 outside of Italy. **** Answers to this question were provided by *n* = 108 respondents from Italy and *n* = 447 outside of Italy.

**Table 2:** Protocols and policies in respondents’ ICUs in Italy and outside of Italy at the time of the survey. The percentages do not sum to 100% as participants could select multiple options.

Visiting Policies	Italy *n* = 118	Outside of Italy *n* = 549
Written visiting policy designed or revised for COVID-19		
Yes	75 (64%)	372 (68%)
Government mandated visiting policy		
No, there are no government mandated restrictions in place	51 (43%)	262 (48%)
Yes, but our ICU has its own policy	24 (20%)	133 (24%)
Yes, and our ICU follows the policy	43 (36%)	154 (28%)
COVID-19-related hospital visiting policy for the hospital wards		
No, the hospital does not restrict visiting for wards	4 (3%)	67 (12%)
Visiting policies in wards are variable and different for each ward of our hospital	17 (14%)	73 (13%)
Yes, and our ICU follows the same policy	61 (52%)	228 (42%)
Yes, and our ICU is more restrictive than hospital policy	18 (15%)	94 (17%)
Yes, and our ICU is less restrictive than the hospital policy	18 (15%)	87 (16%)
ICU visiting policy be changed for specific patients or situations		
Not relevant—no specific policy	6 (5%)	40 (7%)
It requires a written request from the relatives	8 (7%)	45 (8%)
The bedside nurse can make the decision	9 (8%)	51 (9%)
The doctor can make the decision	39 (33%)	261 (48%)
The ICU medical director can make the decision	74 (63%)	218 (40%)
The ICU nursing director can make the decision	20 (17%)	94 (17%)
Hospital hierarchy can make the decision	28 (24%)	102 (19%)
It requires approval at a higher level	1 (1%)	20 (4%)
The ICU visiting policy cannot be changed for specific situations or patients	14 (12%)	60 (11%)
Estimated % difference between set policy and what is offered to relatives *		
0	14 (13%)	85 (18%)
1 to 9	30 (30%)	76 (16%)
10 to 24	32 (30%)	170 (35%)
25 to 49	17 (16%)	78 (16%)
50 or more	14 (13%)	74 (15%)

* Answers to this question were provided by *n* = 107 respondents from Italy and *n* = 483 outside of Italy.

**Table 3:** Communication strategies with relatives in Italy and outside of Italy at the time of the survey. Percentages do not sum up to 100% as participants could select multiple options.

Communication with Relatives of ICU Patients	Italy *n* = 118	Outside of Italy *n* = 549
Do you have an information booklet or webpage?		
No	82 (69%)	300 (55%)
Electronic format only	14 (12%)	108 (20%)
Physical format (booklet)	12 (10%)	110 (20%)
Both (electronic + physical formats)	10 (8%)	31 (6%)
Mode of delivery of general or daily updates		
Updates are given in-person at the bedside (within the limits of visiting)	7 (6%)	136 (25%)
Updates are given in-person, but outside of the clinical area of the ICU	23 (19%)	207 (38%)
Updates are given in-person, but outside of the hospital and outdoors	0	26 (5%)
Updates are given on the phone, on family request	30 (25%)	249 (45%)
Updates are given on the phone; families are called at regular intervals	96 (81%)	257 (47%)
Updates are given via virtual/video conferences	38 (32%)	92 (17%)
Not applicable	0	21 (4%)
Formal meetings or discussions regarding prognosis, treatment plans, or end-of-life care		
Family meetings are held in-person in the same place as before COVID-19	24 (20%)	206 (38%)
Family meetings are held in-person in dedicated area set up since COVID-19	28 (24%)	148 (27%)
Family meetings are held outside of the building, outdoors	6 (5%)	30 (5%)
Family meetings are held via video conference	21 (18%)	82 (15%)
Family meetings are held over the phone	83 (70%)	223 (41%)
Not applicable	5 (4%)	47 (9%)
Which devices are used for virtual visiting?		
Personal devices provided by staff members	22 (19%)	80 (15%)
Personal devices provided by patients or their relatives	30 (25%)	150 (27%)
Computers that are also used for patient care / clinical information systems	4 (3%)	26 (5%)
Devices dedicated to virtual visiting in the ICU	84 (71%)	195 (36%)
Devices repurposed for virtual visiting	5 (4%)	26 (5%)
Not applicable	0	4 (1%)
How is virtual visiting organized?		
Appointments organized by the staff are offered to the relatives on a regular basis	35 (30%)	103 (19%)
Appointments organized by the staff when requested by the doctor or the nurse	37 (31%)	116 (21%)
Appointments organized when requested by the relatives	59 (50%)	164 (30%)
Virtual visiting on request from a relative or the patient (no appointment)	43 (36%)	133 (24%)
Not applicable	4 (3%)	16 (3%)
How frequently do you use virtual visiting? *		
Daily or almost daily for most patients	33 (33%)	78 (24%)
Several times per week for most patients	30 (30%)	96 (30%)
Not more than once a week for most patients	13 (13%)	34 (11%)
Infrequently, only for a few patients	24 (24%)	104 (32%)
Never	1 (1%)	9 (3%)
Do you use virtual / video visiting in your ICU **		
No, virtual visiting is not available	17 (14%)	232 (42%)
Yes, virtual visiting is available, but its use is NOT protocolized	82 (69%)	244 (44%)
Yes, virtual visiting is available, and its use IS protocolized.	19 (16%)	73 (13%)

* Answers to this question were provided by *n* = 101 respondents from Italy and *n* = 321 outside of Italy. ** Answers to this question were provided by *n* = 541 respondents outside of Italy.

## Data Availability

The data presented in this study are available on request from the corresponding author.

## Supplementary Materials

The following supporting information can be downloaded at: https://www.mdpi.com/article/10.3390/healthcare11050669/s1. Table S1: Characteristics of respondent ICUs from Italy over the three study periods; File S1: Original questionnaire.
