# Supplementary material for: Variation in Communication and Family Visiting Policies in Italian Intensive Care Units during the COVID-19 Pandemic: A Secondary Analysis of the COVISIT International Survey
Source: Healthcare (Basel). 2023 Feb 24;11(5):669. doi: 10.3390/healthcare11050669 (PMC10001082; doi:10.3390/healthcare11050669)
Supplement: Supplementary file 1 [file healthcare-11-00669-s001.zip › Appendix S1.pdf]

Data collection tool

Welcome to the COVSIT survey.

Closing ICUs to visitors, either as a blanket rule, or by drastically reducing the number and duration of visits has been used in multiple countries and to various degrees. Most often out of the necessity to protect the patients, their relatives, and an already overstretched health care system.

Multiple reports have described the traumatic separation caused by the inability to visit critically ill loved ones' and the incredible grief of being separated at end of life.

We, as an international group of ICU nurses and doctors, have designed this survey to understand how the COVID-19 pandemic has affected visiting of ICU patients by their relatives and what mitigation strategies may be in place. (click here to access the protocol)

We are seeking one answer only for each ICU. Ideally completed by a senior nurse or doctor that is aware of previous and current visiting policies. *Please discuss with your colleagues to ensure this is done only once.*

We refer to relatives or ICU patients as persons defined by the patient as family, friends, neighbours, relatives, and/or support persons.

The survey investigates 4 core domains:

1. A description of your ICU, including bed availability, staffing and when was the peak of COVID-19 related restrictions to visiting for you (if applicable).
2. Are relatives of ICU patients allowed at the bedside, and if yes for how long?
3. Have you been using videoconferencing or technology to facilitate virtual visiting by relatives of ICU patients?
4. How do you maintain communication with and information to the relatives?

COVISIT has been reviewed as a low and negligible risk project by the Ethics committee of the Royal Brisbane and women's hospital in Australia (LNR/2020/QRBW/71880) and is endorsed by the research committee of the European Society of Intensive Care Medicine.

The survey is translated and available in English, Italian, French, Japanese and Spanish languages, please look for the link at the top right of this page if you wish to change the language.

It will take you 10 to 15 minutes to complete the survey. Please use a computer rather than a phone to record your responses.

The survey is available to departments that define themselves as Intensive Care Units. Usually ICUs provide advance monitoring and organ supportive therapy to critically ill patients. We welcome responses from all departments that treat or do not treat COVID-19 patients

Please click here to complete the survey

Data collection tool

Text in navy blue is not to be displayed and only a guide to build the online data tool.

It is important that we collect one answer for each ICU. Please discuss with your colleagues to ensure this is done only once.

The first 5 questions allow to identify the ICU and remove eventual duplicates. They will be deleted from the database after this step has been completed and prior to analysis. Country information will be kept and analysed as part of the results.

If there are several ICUs with different rules, eg a COVID-19 and a non COVID-19 ICU, please complete the survey several times, one for each ICU

Part 1 – identification of the ICU.

Please identify your role in the ICU as this will help us ensuring there is only one answer per ICU

Q1. ROLE *(will be deleted before analysis)*
(one possible answer)

a) Medical director
b) Medical senior role
c) Nursing Senior or non clinical role
d) Nursing Clinical role
e) Medical other
f) Nursing other
g) Administrative role
h) Other

Q2.COUNTRY *(will be reported as part of the results)*
(list/dropdown)

Q3.Town *(will be deleted before analysis)*
(textfield)

Q4. Name of hospital *(will be deleted before analysis)*
(textfield)

Q5. Name of the ICU *(will be deleted before analysis)*
Only if more than 1 ICU in the hospital, this will be used to avoid duplicates

(textfield)

Data from the following questions will be analysed and reported as the results of the survey.

Q6. Type of Hospital

(one possible answer)

a) Large tertiary teaching hospital, including university hospitals

b) Community based urban hospital

c) Remote / regional hospital

Q7. Funding of the hospital

(multiple possible answers, tickboxes)

Please tick at least one box.

a) Private for-profit hospital

b) Private not-for profit hospital

c) Public hospital

Q8. Number of acute care beds in the hospital (Currently)

(this is the total number of beds in the hospital, including all specialties of medicine, surgery and critical care, but not long term rehabilitation or nursing home beds.)

The following questions ask

1- Is this a Pre-existing ICU or a new ICU

2- when was the peak of COVID-19

With responses we will redirect to a page asking data from before the pandemic, at the peak of the pandemic and currently according to their answers. We will only show what is relevant to the respondent.

Page 2 – Status of the ICU

Q9. Type of ICU

Please read carefully as your choice will determine which questions we ask you later in the survey

(one possible answer, dropdown please)

a) ICU pre-existing to the pandemic and CURRENTLY not managing COVID-19 patients
b) ICU pre-existing to the pandemic and CURRENTLY fully dedicated to COVID-19 patients
c) ICU pre-existing to the pandemic and CURRENTLY managing mixed COVID-19 and non-COVID-19 patients
d) New ICU (built specifically for the pandemic) and CURRENTLY not managing COVID-19 patients.
e) New ICU (built specifically for the pandemic) fully dedicated to COVID-19 patients
f) New ICU (built specifically for the pandemic) and managing mixed COVID-19 and/or non-COVID-19 patients

Page 3 – Timeline

Q10. Timeline: When was the peak of COVID-19 for your ICU ?

(if your ICU has not admitted any COVID-19 patients during the pandemic, please select the month where visiting restriction where at the highest, if not applicable, select NOW.)

(select with list of months from Jan 2020 to March 2021, plus one option on top that says: NOW)

Page 5 ICU Staffing and visiting hours.

Q11. Characteristics of the ICU

please read these instructions carefully:

- **Before the COVID-19 pandemic**: to describe your ICU at baseline, before any restrictions to visiting or change in organisation due to COVID-19. Please enter values for December 2019.

- **At the peak of the pandemic** – When you had the highest number of COVID-19 patients in your ICU.

- **Currently** – use this to describe the current situation. If you are at the peak occupancy indicate it here.

	(column 2)<br>At the Peak of COVID-19	(column 3)<br>Currently
Caseload: % of the patients in the ICU with COVID-19		

Q12. Capacity of the ICU

	(column 1)<br>Before COVID-19 (December 2019)	(column 2)<br>At the Peak of COVID-19	(column 3)<br>Currently
**a. Total number of ICU beds (actual capacity)**			

Q13 Staffing of the ICU

	(column 1)<br>Before COVID-19 (December 2019)	(column 2)<br>At the Peak of COVID-19	(column 3)<br>Currently
c. Nurse patient ratio: 1 nurse cares for... patients			
c. Senior Doctor patient ratio: 1 Dr. cares for... patients			
d. Junior Doctor patient ratio: 1 Dr. cares for... patients			

Q14. Routine in-person Visiting conditions for ICU patients with COVID-19.

Please let us know if physical persons are allowed at the bedside of the patient, or if they can only view their relatives through a window or if there are no visitors in the ICU or no visitors in the hospital.

	(column 2)<br>At the Peak of COVID-19	(column 3)<br>Currently
Response items may be changed but can't become longer than what they are now	- At the bedside<br>- View through a window<br>- No visitors in the ICU<br>- No visitors in the Hospital	

Q15. In-person Visiting hours per day, at the bedside, for ICU patients with COVID-19

	At the Peak of COVID-19	Currently
	- Unrestricted visiting hours<br>- No in-person visiting<br>- less than 30 minutes<br>- 30 to 60 minutes<br>- 1 hour<br>- 2 hours<br>- 3 hours<br>- Etc up to 24h	

Q16. Specific reasons where visiting policies for patients with COVID-19 may be different and more liberal

Please chose the reasons why visitors may be allowed or allowed for longer time periods despite restrictions.

		At the Peak of COVID-19	Currently
New admission			
Clinical deterioration			
End of Life		Increased Visiting<br>No change	
Conscious patient			
On family request			
When no longer considered contagious			
Other			

Q17. is the visiting policy different for patients WITHOUT COVID-19

If visiting policies are different for patients with and without COVID-19, please enter how many hours per day visitors are allowed at the bedside of patients without COVID-19

(column 1)<br>Before COVID-19 (December 2019)	(column 2)<br>At the Peak of COVID-19	(column 3)<br>Currently

	- Same as for COVID-19 pts<br>- Unrestricted visiting hours<br>- No in-person visiting<br>- less than 30 minutes<br>- 30 to 60 minutes<br>- 1 hour<br>- 2 hours<br>- 3 hours<br>- Etc up to 24h	same

From here, for simplicity, we will only ask the questions as of NOW/CURRENTLY.

Page 9 Visiting Policies for ICU patients

From this point of the survey, please answer to describe your current situation. As of today.

Q18. Do you have a WRITTEN policy for visiting that was designed or revised to include COVID-19 specifics ? (one possible answer, dropdown menu)

- NO
- YES

Q19. Is there a government mandated visiting policy currently (city, state or country restrictions to hospital or ICU visiting due to COVID-19). (one possible answer, dropdown menu)

- NO, there are NO government mandated restrictions in place.
- Yes, the government restricts visiting in hospitals, our ICU has its own policy.
- Yes, the government restricts visiting in hospitals and our ICU follows the policy.

Q20 Is there a COVID-19 related hospital visiting policy for the hospital wards (above the government policy, if any)? (one possible answer, dropdown menu)

- NO, the hospital does not restrict visiting for the wards.
- Visiting policies in the wards are variable and different for each ward of our hospital
- Yes, the hospital restricts visiting for the wards, and our ICU follows the same policy.
- Yes, the hospital restricts visiting for the wards, and our ICU IS MORE restrictive than the hospital policy.
- Yes, the hospital restricts visiting for the wards, but our ICU IS LESS restrictive than the hospital policy.

Q21 Can the ICU visiting policy be changed for specific patients or situations?

(eg, End of life, family request, compassionate grounds, if not clearly specified in the policy)

(multiple possible answers, use checkboxes)

- Not relevant, we don't have a specific policy during the pandemic
- Yes, it a requires written request from the relatives
- Yes, the bedside nurse can make the decision
- Yes, the doctor can make the decision
- Yes, the director of the ICU can make the decision
- Yes, the nursing director of the ICU can make the decision
- Yes, hospital hierarchy can make the decision
- Yes, requires approval at a higher level (eg ministerial)
- No, ICU visiting policy cannot be changed for any specific situation or patients

Q22 Please estimate the variability between the set policy you have described and what is offered to relatives of ICU patients:
Please enter an estimated percentage (%) cases for which visiting has been more liberal than the set policy and relatives were allowed at the bedside more often or for longer periods.
-----Textfield, allow numbers up to 100 ----

Page 10 communication with relatives of ICU patients

These questions refer to the current organisation of your ICU

Q23 COVID-19 ICU Information booklet
Do you have an information booklet or webpage with information on COVID-19, visiting and PPE for relatives of ICU patients? (multiple possible answers)

a) No
b) Yes, electronic format (webpage or similar)
c) Yes, physical booklet

Q24. General or daily updates
This refers to general updates on the status of the patient, please refer to what has been happening in the days prior to answering the survey. (multiple possible answers)

a) Updates are given in person at the BEDSIDE (within the limits of visiting)
b) Updates are given in person, but OUTSIDE of the CLINICAL AREA OF the ICU.
c) Updates are given in person, but OUTSIDE of the HOSPITAL and OUTDOORS.
d) Updates are given on the phone, on family's request
e) Updates are given on the phone; families are called at regular intervals by ICU staff
f) Updates are given via virtual/video-conferences.
g) Not applicable

Q25. Formal meetings or discussions regarding prognosis, treatment plans or End Of Life
(multiple possible answers)

a) Family meetings are held in person in the same place as before COVID-19
b) Family meetings are held in person but in an area dedicated to meetings setup since COVID-19
c) Family meetings are held OUTSIDE of THE BUILDING, OUTDOORS
d) Family meetings are held via VIDEO CONFERENCE.
e) Family meetings are held over the PHONE.
f) Not applicable

Q26. Do you use virtual / video visiting in your ICU.
Do you offer the possibility for relatives of ICU patients to use Video-conferencing or Video-chat to virtually visit their family members when in-person visiting is not possible?
(branching : if YES > send to q26 Which devices, if NO > send to Q30)

- No, virtual visiting is not available.
- Yes, virtual visiting is available, but its use is NOT protocolized.
- Yes, virtual visiting is available, and its use IS protocolized.

Q27. Which devices are used for virtual visiting
(multiple possible answers)

- Personal devices provided by staff members.
- Personal devices provided by patients or their relatives.
- Computers that are also used for patient care / clinical information systems

- Devices dedicated to virtual visiting (eg Tablets, computers) in the ICU (not used for something else)
- Devices usually dedicated to virtual clinical rounds repurposed for virtual visiting
- Not applicable

Q28. How is virtual visiting organized?
(multiple possible answers)

- Appointments Organized by the staff are offered to the relatives on a regular basis
- Appointments Organized by the staff when requested by the doctor or the nurse
- Appointments Organized when requested by the relatives
- Virtual visiting can be initiated on request from a relative or the patient (no appointment).
- Not applicable

Q29. How frequently do you use virtual visiting?

(One possible answer)

- Daily or almost daily for most patients
- Several times per week for most patients
- Not more than once a week for most patients
- Infrequently, only for a few patients
- Never

Q30 Please rank the following reasons why relatives did not or could not visit their loved ones in ICU while visiting was in theory possible.
Please tick how frequently relatives have not visited for each potential reason below. You may use the textbox of the following question to give some details if you whish.
5 point Likert scale matrix (never/rarely/at least once a week/at least once a day/not applicable)

- Fear from catching COVID-19
- Own COVID-19 illness
- Fear from disturbing clinical care
- Fear from being overwhelmed by ICU
- Inability to travel due to lockdown
- Inability to enter the hospital

Q31: Would you please share with the investigators your perspectives/ experience with caring for families of COVID 19 patients?

Q32. Do you certify this data to be complete and accurate and would you like your response to be recorded.
a) yes, my response is complete and accurate and should be recorded
b) no, I was just testing the survey, do not record my response
c) no, do not record this response (other reasons)
