# Supplementary material for: Variation in Communication and Family Visiting Policies in Italian Intensive Care Units during the COVID-19 Pandemic: A Secondary Analysis of the COVISIT International Survey
Source: Healthcare (Basel). 2023 Feb 24;11(5):669. doi: 10.3390/healthcare11050669 (PMC10001082; doi:10.3390/healthcare11050669)
Supplement: Supplementary file 1 [file healthcare-11-00669-s001.zip › Table S1.pdf]

Article

Variation in Communication and Family Visiting Policies in Italian Intensive Care Units during the COVID-19 Pandemic: A Secondary Analysis of the COVISIT International Survey

Barbara Simone [1], Mariachiara Ippolito [1,2], Pasquale Iozzo [2], Francesco Zuccaro [3], Antonino Giarratano [1,2], Maurizio Cecconi [4,5], Alexis Tabah [6,7,8] and Andrea Cortegiani [1,2,*]

[1] Department of Surgical, Oncological and Oral Science, University of Palermo, Via Liborio Giuffrè 5, 90127 Palermo, Italy
[2] Department of Anaesthesia, Intensive Care and Emergency, University Hospital Policlinico Paolo Giaccone, 90127 Palermo, Italy
[3] Department of Anesthesia and Intensive Care, Ospedale Madonna delle Grazie, Azienda Sanitaria Matera, 75100 Matera, Italy
[4] Department of Biomedical Sciences, Humanitas University, Via Rita Levi Montalcini 4, Pieve Emanuele, 20072 Milan, Italy
[5] Department of Anesthesia and Intensive Care, IRCCS Humanitas Research Hospital, Via Manzoni 56, Rozzano, 20089 Milan, Italy
[6] Intensive Care Unit, Redcliffe Hospital, Metro North Hospital and Health Services, Redcliffe, QLD 4020, Australia
[7] School of Clinical Sciences, Queensland University of Technology, Brisbane, QLD 4000, Australia
[8] Antimicrobial Optimisation Group, UQ Centre for Clinical Research, The University of Queensland, Brisbane, QLD 4029, Australia
* Correspondence: andrea.cortegiani@unipa.it; Tel.: +39-0916552751

Table S1. Characteristics of respondent ICUs from Italy over the three study periods.

Characteristics	*Before COVID-19 n (%)*	*At Peak*	*At time of survey*
ICU patients with COVID-19 (%)		n= 116	n= 117
Less than 25	-	42 (36%)	50 (43%)
25 to 49	-	4 (3%)	4 (3%)
50 to 89	-	9 (8%)	21 (18%)
90 or more	-	61 (53%)	42 (36%)
Total number of ICU beds	n=88	n=118	n= 117
1 to 8	42 (48%)	19 (16%)	29 (25%)
9 to 16	36(41%)	42 (36%)	47 (40%)
17 to 24	5 (6%)	30 (25%)	22 (19%)
25 to 40	3(3%)	19 (16%)	12 (10%)
More than 40	2 (2%)	8 (7%)	7 (6%)
Senior doctor to patient ratio	n=81	n=108	n= 105
Less than 1:6	41 (51%)	38 (35%)	41 (39%)
1:6 to 1:10	40 (49%)	57(53%)	54 (51%)
More than 1:10	0 (0%)	13 (12%)	10 (10%)
Junior doctor-to-patient ratio	n=46	n=76	n= 76
Less than 1:6	26 (57%)	36 (47%)	33 (43%)
1:6 to 1:10	20 (43%)	32 (42%)	34 (45%)
More than 1:10	0 (0%)	8 (11%)	9 (12%)

Nurse-to-patient ratio	n=80	n=110	n= 108
1:1	0 (0%)	0 (0%)	1 (1%)
1:2	54 (68%)	52 (47%)	74 (69%)
1:3	22 (28%)	43 (39%)	27 (25%)
More than 1:3	4 (5%)	15 (14%)	6 (6%)
